# Adipose-derived stem cells alleviate liver apoptosis induced by ischemia-reperfusion and laparoscopic hepatectomy in swine

**DOI:** 10.1038/s41598-018-34939-x

**Published:** 2018-11-15

**Authors:** Yansong Ge, Qianzhen Zhang, Hui Li, Ge Bai, Zhihui Jiao, Hongbin Wang

**Affiliations:** College of Veterinary Medicine, Northeast Agricultural University, Harbin, 150030 P.R. China

**Keywords:** Adipose-derived Stem Cells (ADSCs), Laparoscopic Hepatectomy, ADSCs Group, Bama Miniature Pigs, ADSCs Treatment, Single-molecule biophysics, Mesenchymal stem cells

## Abstract

Hepatic ischemia-reperfusion (I/R) injury is inevitable during hepatectomy and may cause both postoperative morbidity and mortality. Regenerative medicine suggested adipose-derived stem cells (ADSCs) as an attractive tool for the treatment of liver diseases. In this study, we investigated the effect of ADSCs in an I/R model combined with laparoscopic hepatectomy in swine. Eighteen Bama miniature pigs were randomly divided into Sham, IRI, and ADSCs groups. ADSCs (1 × 10^6^/kg) were injected through liver parenchyma immediately after hemihepatectomy. The apoptosis-related role of ADSCs was studied. The results showed that ADSCs transplantation reduced both pathological and ultrastructural changes and decreased the number of apoptotic-positive cells. In the ADSCs group, Fas, Fas ligand (FasL) protein, and mRNA were downregulated and the enzyme activities of Caspase3, Caspase8, and Caspase9 were significantly decreased. In addition, ADSC therapy significantly increased the ratio of Bcl-2/Bax protein and mRNA compared to the IRI group. In conclusion, ADSCs attenuated both I/R and hepatectomy-induced liver apoptosis in a porcine model, and offers a potential therapeutic option for hepatic I/R and hepatectomy.

## Introduction

Gagner *et al*. and Reich *et al*. reported the first laparoscopic hepatectomy during the early 1990s^1,2^. With the ongoing development of both laparoscopic technology and equipment, the application of laparoscopy in liver surgery has increased, demonstrating its feasibility. Laparoscopic liver resection offers the advantages of reduced blood loss and transfusion requirements, rapid postoperative recovery, and decreased amount of complications compared to lobectomy^3–5^. I/R injury is an inevitable problem during hepatectomy and ischemia leads to the inhibition of oxidative phosphorylation, thus decreasing both ATP synthesis and intracellular pH levels. This may cause intracellular and mitochondrial calcium overload, inducing endoplasmic reticulum stress and necrosis. Although reperfusion can restore the supply of both nutrients and oxygen, it also increases ROS production; furthermore, inflammatory cells infiltrate ischemia tissues, which promotes apoptosis and aggravates I/R injury^6,7^. However, only few animal models have been used for simultaneous I/R and hepatectomy injury, which is necessary to investigate a protective strategy in a model that combines I/R with hepatectomy^8–11^.

ADSCs have been shown to be abundantly organized, easily harvested, and to possess high self-renewing ability. Moreover, ethical issues related to embryonic stem cells and induced pluripotent stem cells do not apply for ADSCs^12,13^. Zuk *et al*. first demonstrated that ADSCs have multiple differentiation potentials^14^. In 2005, Seo *et al*. first reported that ADSCs have the potential to differentiate into hepatocytes^15^. After that, ADSCs have been used to treat various liver diseases, such as hepatic failure, concanavalin A-induced hepatitis, and liver fibrosis^16–18^. Further studies have shown that ADSCs can reduce the process of apoptosis by the paracrine^19,20^. However, only few studies investigated ADSC protection in large animals. To investigate a prospective link to human ADSC treatment of liver diseases, here, we investigated the anti-apoptotic effects of ADSCs in an I/R model combined with laparoscopic hepatectomy using a swine model.

## Materials and Methods

### Animals

Eighteen clinically healthy Bama miniature pigs were provided by the Bama Miniature Pig Farm of the College of Life Sciences (Harbin, China). All animals were housed in the animal facility and subjected to standard conditions. The study protocol was approved by the Animal Care and Use Committee of the Northeast Agricultural University (approved by the State Council on October 31^st^, 1988 and promulgated by Decree No. 2 of the State Science and Technology Commission on November 14^th^, 1988). All methods were performed in accordance with these approved guidelines.

### Isolation and Characterization of ADSCs

Subcutaneous adipose tissue of the abdomen was obtained from Bama miniature pigs. The adipose tissue was minced, washed, and digested with 0.01% collagenase type I for 45 min at 37 °C under gentle agitation; serum-containing culture (DMEM with 10% FBS) was added to terminate the digestion. The tissue was filtered through a 75-μm cell strainer to remove debris, which was followed by centrifugation at 1500 rpm for 10 min. The tissue was incubated with an erythrocyte lysing reagent (Solarbio, China) for 5 min and washed twice with DMEM (HyClone, USA). Cells were suspended with L-DMEM containing 10% FBS (Clark, USA), 2 mM L-glutamine, 100 μg/mL penicillin, and 100 μg/mL streptomycin (all obtained from Solarbio, China). Then, cells were seeded at a density of 1 × 10^6^/mL and cultured at 37 °C in a 5% CO_2_ humidified incubator (Galaxy 170 S, Eppendorf, Germany).

Passage 3–5 ADSCs were characterized via osteogenic, adipogenic, and hepatic differentiation using differentiation media (Cyagen Biosciences, USA). Osteogenic differentiation was labeled using 0.1 mg/mL Alizarin Red staining of the mineralized matrix, adipogenic differentiation was labeled using 0.5% Oil Red O staining of the lipid droplets, and hepatic differentiation was labeled using PAS solution staining of glycogen (Solarbio, China). Passage 3–5 ADSCs were used for phenotypic characterization, ADSCs were labeled for 60 min using anti-porcine FITC-CD29, FITC-CD34, FITC-CD44, and FITC-CD105 (1:1000, Abcam, USA). Cells were washed twice and suspended with PBS. ADSCs were acquired via flow cytometry and analyzed with FACSDiva software (BD, USA).

### Surgical Procedure

Eighteen miniature pigs were randomly divided into a Sham group, an IRI group, and an ADSCs group (six animals per group). In the Sham group, only the pneumoperitoneum was established and the liver lobe was flipped. Both the IRI group and the ADSCs group underwent left hemihepatectomy as previously described after right hepatic ischemia for 60 min^21^. The ADSCs group received one ADSC injection (1 × 10^6^/kg) through the liver parenchyma immediately after hemihepatectomy. Blood samples and liver tissues were preoperatively collected as well as at postoperative 1 d, 3 d, and 7 d.

### Adenovirus Transfection of Green Fluorescent Protein (GFP) and Liver Bioluminescence Imaging

Adenovirus particles carrying the GFP gene (Hanbio, China) were added to the passage 2–3 ADSCs culture medium (MOI is 20:1). After incubation for 24 h, the culture medium was replaced with fresh culture medium. The transfection efficiency of GFP in ADSCs was assessed via fluorescence microscopy. Liver bioluminescence imaging was performed to detect pig livers on 1 d after surgery using the Tanon 5200 system (Tanon, China).

### Immunohistochemical Analysis

Tissue samples were fixed in 4% paraformaldehyde for 24 h and then dehydrated, cleared, and embedded in paraffin. 4-μm paraffin sections were blocked in H_2_O_2_ for 10 min and heated in a microwave for 10 min. BSA was added and then, the sections were incubated with anti-Fas and anti-FasL (1:200, Sangon Biotech, China) overnight at 4 °C. Then, the sections were washed twice with PBS and incubated with biotin-labeled anti-IgG (Boster, China). Furthermore, the sections were incubated with streptavidin-labeled HRP for 20 min at room temperature and then visualized with DAB. The sections were counterstained with hematoxylin and coverslipped with neutral balsam. All sections were quantified with IPP 6.0 software (Media Cybernetics, USA).

### Histological Analysis

Liver samples were fixed in 4% paraformaldehyde for 24 h, then conventionally dehydrated, cleared, and embedded in paraffin. Samples were sliced to 4 μm and stained with both hematoxylin and eosin. Then, the sections were treated with acidic alcohol, washed under running water, and stained with eosin. After coverslipping with neutral balsam, the sections were evaluated via light microscopic examination.

### Electron Microscopy

Liver tissues were cut into small pieces, fixed with 2.5% glutaraldehyde, and then post-fixed in OxO_4_. After dehydration with ethanol, the specimens were embedded in epoxy resin and then sectioned extra thin. The sections were stained with both lead citrate and uranyl acetate and then observed using an H-7650 electron microscope (Hitachi, Japan).

### TUNEL Assay

4-μm sections were evaluated via the TUNEL assay using an apoptosis detection kit (Roche, Germany) according to the manufacturer’s instructions. TUNEL-positive cells were examined via light microscope.

### Caspase Activity Assay

Caspase3, Caspase8, and Caspase9 activities in liver tissues were determined via Caspase Activity Assay Kits (Beyotime, China) according to the manufacturer’s instructions.

### Western Blot

Extraction of total protein from liver tissue samples was conducted via the Total Protein Extraction Kit (Beyotime, China); then, the protein concentration was detected using a BCA Kit (Beyotime, China). An equal amount of protein (30 μg) was separated via 12% SOD-PAGE and electroblotted onto nitrocellulose membranes (Biosharp, China). Then, the membranes were blocked with 5% nonfat milk for 2 h and incubated with both anti-Bcl-2 and anti-Bax primary antibodies (1:500) at 4 °C overnight. After washing with TBST, the blot was incubated with HRP-conjugated anti-IgG for 2 h and detected using an enhanced ECL reagent (Biosharp, China) on an AI600 System (GE Healthcare, UK). Relative protein expression was quantified via ImageJ software and normalized against the β-Actin control (1:2000, Sangon Biotech, China).

### RT-PCR and qRT-PCR Analyses

Total RNA was extracted from liver tissue using the TRIzol reagent (Invitrogen, China) and then, cDNA was generated using the ReverTra Ace qPCR RT Master Mix (Toyobo, Japan). Detection of hepatocyte-specific genes in liver and ADSCs was conducted by RT-PCR reactions using a PCR thermal cycler (BIO-RAD, USA). qTR-PCR was performed on a LightCycler 480 System (Roche, Germany) in a 10-μL final reaction volume containing 5 μL SYBR Green Master (Rox), 1 μL cDNA, 3.4 μL ddH_2_O, and 0.3 μL of each primer. The program was as follows: 15 s at 95 °C; 40 cycles of 15 s at 95 °C, 60 s at 60 °C, and 30 s at 60 °C. The primers (Table 1) were synthesized by Sangon Biotech (Shanghai, China).

**Table 1:** Primers used for both RT-PCR and qRT-PCR.

Gene	Primer sequence
ALB	Forward 5′-GCACGAGAAGACACCAGTGAGC-3′ Reverse 5′-GGTCTGCATGGAAGGTGAAGGTTC-3′
TAT	Forward 5′-GCTTCCTCAAGACCAACGCTGAC-3′ Reverse 5′-CAACCAACCGCTCTGTGAACTCC-3′
HNF4	Forward 5′-TCGGAGGAGTGTGAGGAAGAACC-3′ Reverse 5′-GCTTGACCTGCGAGTGCTGATC-3′
Bcl-2	Forward 5′-GAGGATTGTGGCCTTCTTTG-3′ Reverse 5′-GCCGGTTCAGGTACTCAGTC-3′
Bax	Forward 5′-TTCAGGGTTTCATCCAGGATCG-3′ Reverse 5′-ATCCTCTGCAGCTCCATGTTAC-3′
Fas	Forward 5′-TGTCCGGGATCTGGGTTCTC-3′ Reverse 5′-GGCATGGCTGACAGCAGAAT-3′
FasL	Forward 5′-CTTCATGGTTCTGGTGGCCC-3′ Reverse 5′-CTGTATGCCTTTGGCTGGCA-3′
β-actin	Forward 5′-TCTGGCAACCACACCTTCT-3′ Reverse 5′-TGATCTGGGTCATCTTCTCAC-3′

### Statistical Analysis

All data are expressed as mean ± SD. GraphPad Prism 7 (GraphPad Software, USA) was used to process all data. Statistical differences within each group were identified via ANOVA test with an additional Turkey’s post hoc test.

## Results

### Characteristics of ADSCs

**Figure 1:**
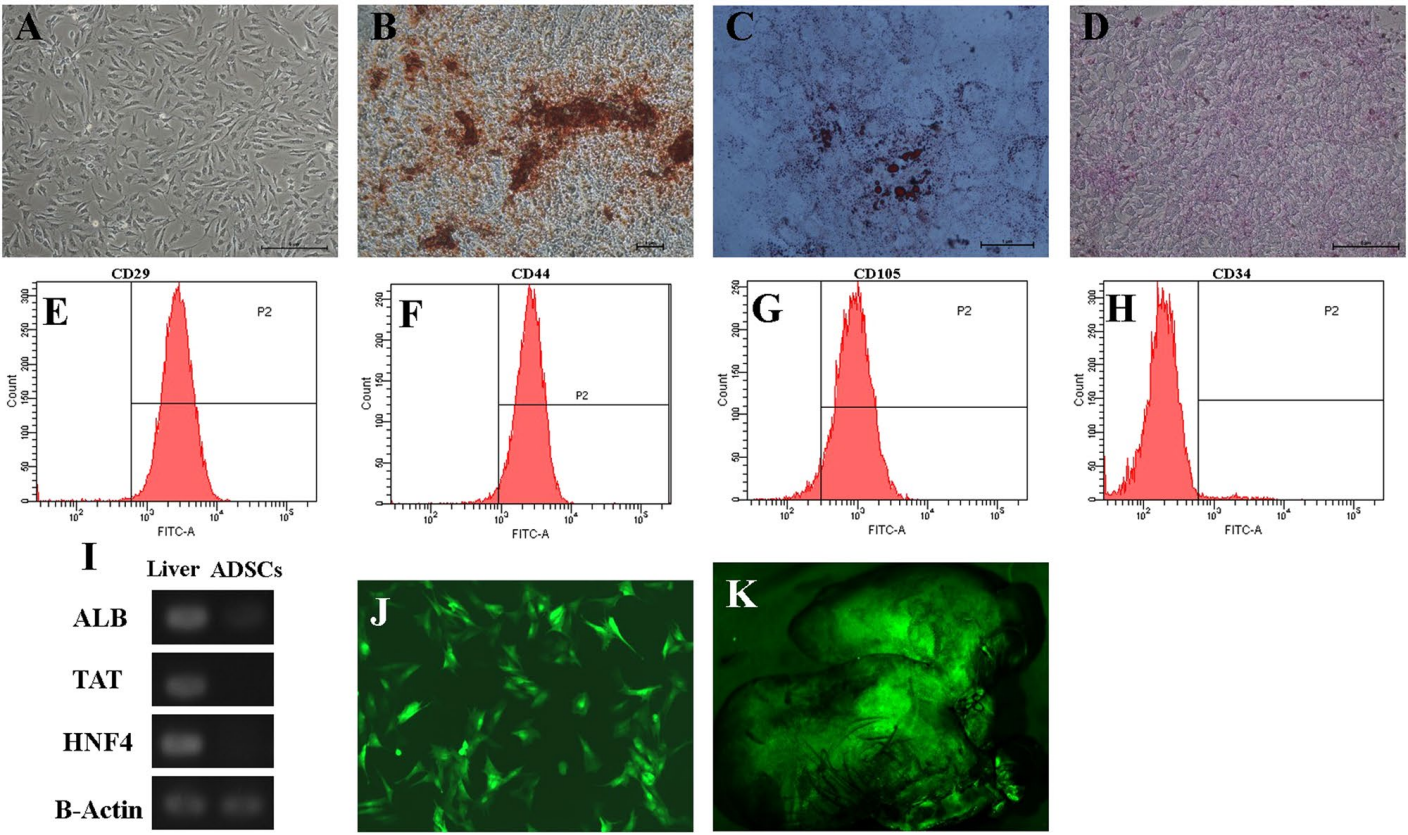
Characteristics of ADSCs and bioluminescence imaging. (**A**) Passage three of ADSCs cultured *in vitro* (magnification × 100). (**B**) Osteogenic differentiations of ADSCs were labeled with Alizarin Red (magnification × 200). (**C**) Adipogenic differentiations of ADSCs were labeled with Oil Red O (magnification × 400). (**D**) Hepatic differentiations of ADSCs were labeled with PAS (magnification × 100). (**E**–**H**) Flow cytometry analysis demonstrated that ADSCs were positive for CD29, CD44, and CD105 and negative for CD34. (**I**) Expression of the liver-specific genes ALB, TAT, and HNF4 in liver and ADSCs. (**J**) Adenovirus Transfection of GFP. (**K**) Liver Bioluminescence Imaging.

Porcine ADSCs had a fibroblast-like spindle shape (Fig. 1A) and rapidly proliferated in the culture medium. Multiple differentiation potentials towards osteogenic, adipogenic, and hepatic were confirmed *in vitro*. Alizarin Red staining showed the generation of calcium crystals (Fig. 1B), Oil Red O staining showed the generation of lipid droplets (Fig. 1C), and PAS staining showed the generation of glycogen (Fig. 1D). Investigation of ADSCs via flow cytometry yielded positive for CD29 (99.1%), CD44 (97.4%), and CD105 (95.3%) and negative for CD34 (0.2%) (Fig. 1E-H). The expression of liver-specific genes such albumin (ALB), tyrosine aminotransferase (TAT), and hepatocyte nuclear factor 4 (HNF4) were not detected in passage 3 ADSCs; liver tissue was used as a positive control (Fig. 1I).

### Fate of ADSCs in Liver

The results showed that GFP could be clearly observed by fluorescence microscopy after transfection with adenovirus particles (Fig. 1J) and the transfection efficiency exceeded 90%. In addition, 24 h after transplantation, GFP-carrying ADSCs were observed in the right lobes of the liver via bioluminescence imaging (Fig. 1K), indicating that the transplantation of ADSCs was successful and ADSCs survived in the liver.

### Effects of ADSCs on Histopathology and Ultrastructure Changes

**Figure 2:**
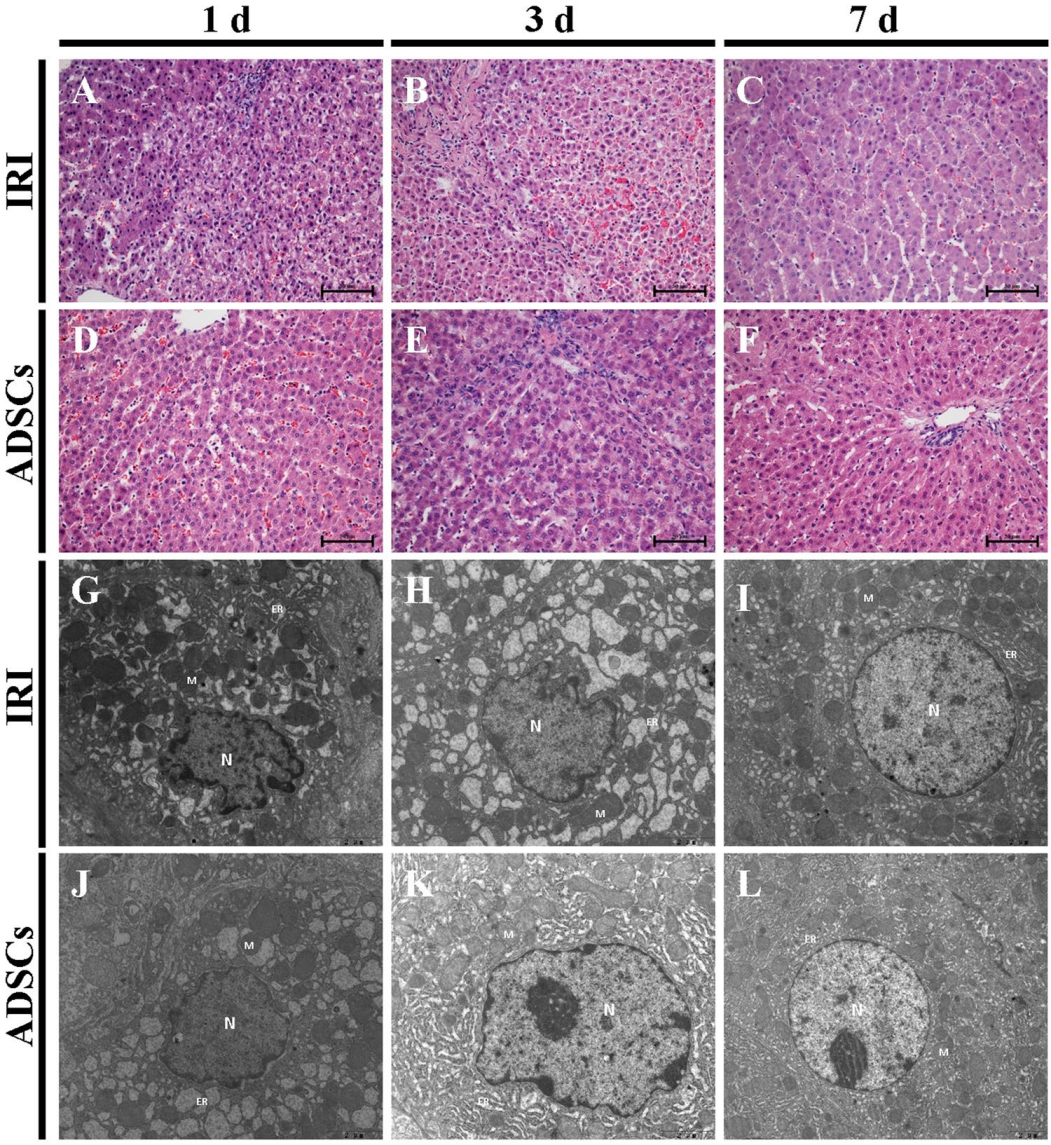
Histopathologic and ultrastructural changes in the liver. Hematoxylin and cosin staining, (**A**–**C**) IRI group, 1 d, 3 d, and 7 d. (**D–F**) ADSCs group, 1 d, 3 d, and 7 d (magnification × 400). Electron microscopy analysis, (**G**–**I**) IRI group, 1 d, 3 d, and 7 d. (**J–L**) ADSCs group, 1 d, 3 d, and 7 d (magnification, G-K × 12000, L × 8000).

Histological analysis showed that at 1 d post operation, multiple focal necroses, severe vacuolar degeneration, and a large number of inflammatory cells infiltrating the liver tissue were observed in the IRI group (Fig. 2A). Inflammatory cell infiltration and hepatic vacuolar degeneration began to decrease on 3 d (Fig. 2B) and minimal inflammatory cell infiltration and slight vacuolar degeneration were observed on 7 d (Fig. 2C). In contrast, histological changes following ADSCs treatment included the reduction of vacuolar degeneration and necrosis on 1 d (Fig. 2D). Only a small number of inflammatory cells infiltrated the liver tissue on 3 d (Fig. 2E) and both the portal region and hepatic lobule recovered to normal (Fig. 2F).

Electron microscopic examination showed nuclear membrane shrinkage, chromatin condensation, mitochondria swelling, and strong endoplasmic reticulum expansion; these effects were observed in the IRI group on 1 d (Fig. 2G). Nuclear membrane shrinkage and chromatin condensation began to decrease on 3 d (Fig. 2H) and a slight expansion of the endoplasmic reticulum was observed on 7 d (Fig. 2I). After treatment with ADSCs, changes in nuclear membrane shrinkage, swelling of mitochondria, and expansion of the endoplasmic reticulum were alleviated between 1 d to 7 d compared to the IRI group (Fig. 2J-L). These results suggest that ADSCs treatment reduced hepatic injury, inflammatory cell infiltration, and ultrastructure changes against I/R and hepatectomy injury.

### Hepatocyte Apoptosis Rate

**Figure 3:**
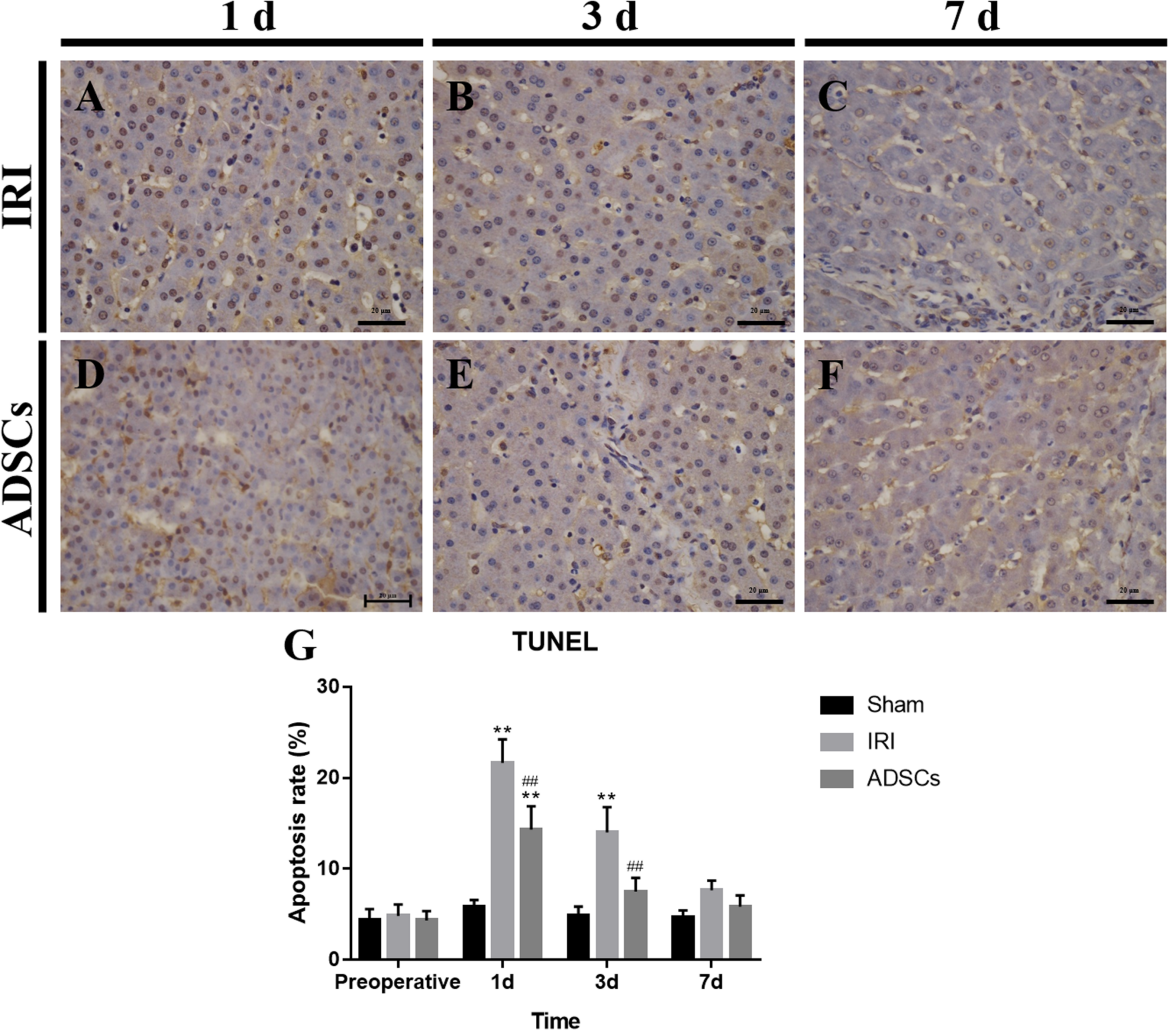
TUNEL staining for liver tissues. (**A**–**C**) IRI group, 1 d, 3 d, and 7 d. (**D**–**F**) ADSCs group, 1 d, 3 d, and 7 d (magnification × 400). (**G**) Apoptosis rate of hepatocytes. **P < 0.01, vs. sham group, ^##^P < 0.01, vs. IRI group.

Figure 3 shows the results of TUNEL staining. The ratio of TUNEL-positive cells peaked on 1 d and then decreased on 3 d in the IRI group (p < 0.01). A significant decrease of positive cells was observed in the ADSCs group between 1 d to 3 d compared to the IRI group (p < 0.01) (Fig. 3G). The data indicate that ADSCs treatment decreases the rate of apoptosis.

### Effects of ADSCs on Caspase activities

**Figure 4:**
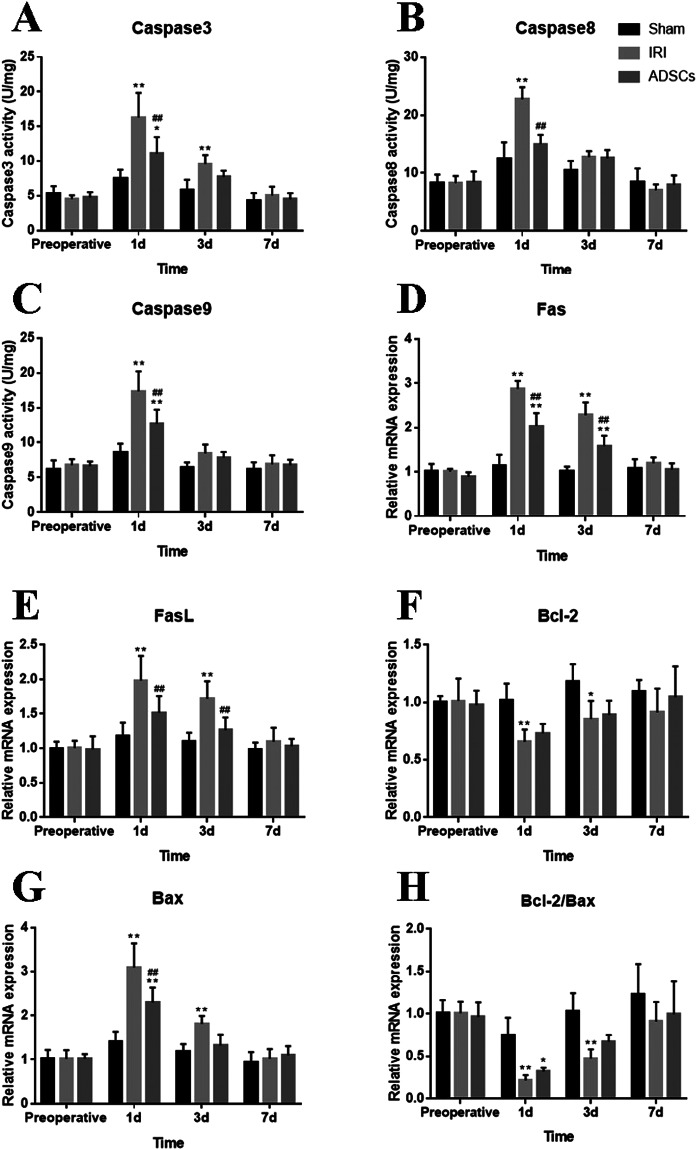
Caspase activities and apoptosis gene expression in liver tissues. (**A**–**C**) Caspase3, Caspase8, and Caspase9 activities in liver tissues. (**D**–**H**) Apoptosis gene expression in liver tissues. *P < 0.05, vs. sham group, **P < 0.01, vs. sham group, ^##^P < 0.01, vs. IRI group.

Liver activities of caspase were analyzed following both I/R and hepatectomy. The Caspase3 activity peaked on 1 d in both the IRI group (p < 0.01) and the ADSCs group (p < 0.05), while ADSCs treatment significantly reduced the level of Caspase3 compared to the IRI group. Caspase3 activity still remained significantly higher on 3 d in the IRI group than in the Sham group (p < 0.01) (Fig. 4A). Caspase8 activity markedly increased on 1 d compared to both the IRI and the ADSCs groups (p < 0.01) (Fig. 4B). Caspase9 levels increased in both the IRI and the ADSCs groups on 1 d (p < 0.01). After ADSCs treatment, the Caspase9 activity significantly decreased compared to the IRI group (p < 0.01) (Fig. 4C).

### Effects of ADSCs on Apoptosis Genes

Apoptosis genes were measured via qRT-PCR. Fas expression increased significantly during 1 d to 3 d in both the IRI and the ADSCs groups (p < 0.01). Similarly, FasL expression markedly increased on 1 d and 3 d in the IRI group (p < 0.01). Compared to the IRI group, Fas and FasL mRNA were significantly decreased in response to ADSCs treatment on 1 d and 3 d (p < 0.01) (Fig. 4D and E). The expression of Bcl-2 mRNA in liver tissue was significantly decreased in the IRI group on 1 d (p < 0.01) and 3 d (p < 0.05). Treatment with ADSCs decreased Bcl-2 mRNA expression; however, no significant difference was found between the other two groups (Fig. 4F). A significant increase of Bax mRNA was observed on 1 d and 3 d in the IRI group (p < 0.01). Bax levels increased significantly on 1 d in the ADSCs group compared to the Sham group; however, ADSCs treatment significantly decreased Bax expression compared to the IRI group (p < 0.01) (Fig. 4G). Additionally, both I/R and hepatectomy induced sharp decreases of the Bcl-2/Bax ratio on 1 d and 3 d (p < 0.01), the Bcl-2/Bax ratio also decreased significantly on 1 d (p < 0.05); however, no significant difference was found between the IRI group and the ADSCs group (Fig. 4H). These results indicate that ADSCs suppress the expression of proapoptotic genes.

### Effects of ADSCs on Apoptosis Proteins

**Figure 5:**
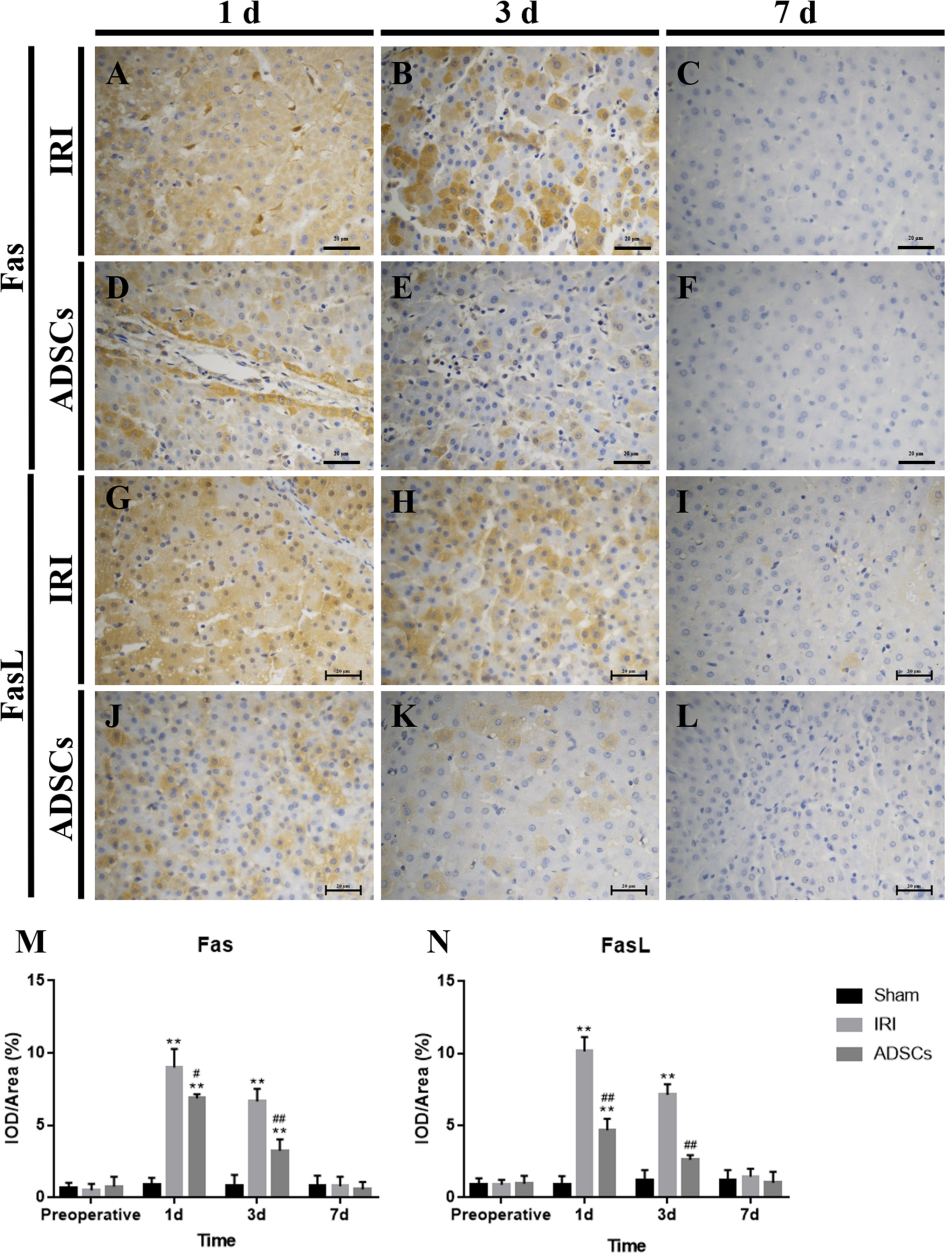
Fas and FasL immunohistochemistry staining of liver tissues. (**A**–**C**) Fas immunohistochemistry staining of the IRI group, 1 d, 3 d, and 7 d. (**D**–**F**) Fas immunohistochemistry staining of the ADSCs group, 1 d, 3 d, and 7 d. (**G**–**I**) FasL immunohistochemistry staining of the IRI group, 1 d, 3 d, and 7 d. (**J**–**L**) FasL immunohistochemistry staining of the ADSCs group, 1 d, 3 d, and 7 d (magnification × 400). (**M** and **N**) Fas and FasL expressions in liver tissues. **P < 0.01, vs. sham group, ^#^P < 0.05, vs. IRI group, ^##^P < 0.01, vs. IRI group.

Immunohistochemistry showed Fas and FasL protein expressions in liver tissues (Fig. 5A–L). Cytoplasmic expression of Fas was significantly upregulated during 1 d and 3 d in both the IRI and the ADSCs groups (p < 0.01). After ADSCs treatment, Fas expression was significantly reduced on 1 d (p < 0.05) and 3 d (p < 0.01) (Fig. 5M). Similarly, FasL protein was significantly elevated on 1 d and 3 d in the IRI group (p < 0.01). The expression of FasL in the ADSC group was significantly higher than in the Sham group on 1 d (p < 0.01) and significantly lower than that in the IRI group during 1 d and 3 d (p < 0.01) (Fig. 5N). These results suggest that ADSCs may inhibit the expression of cytoplasmic Fas and FasL protein.

**Figure 6:**
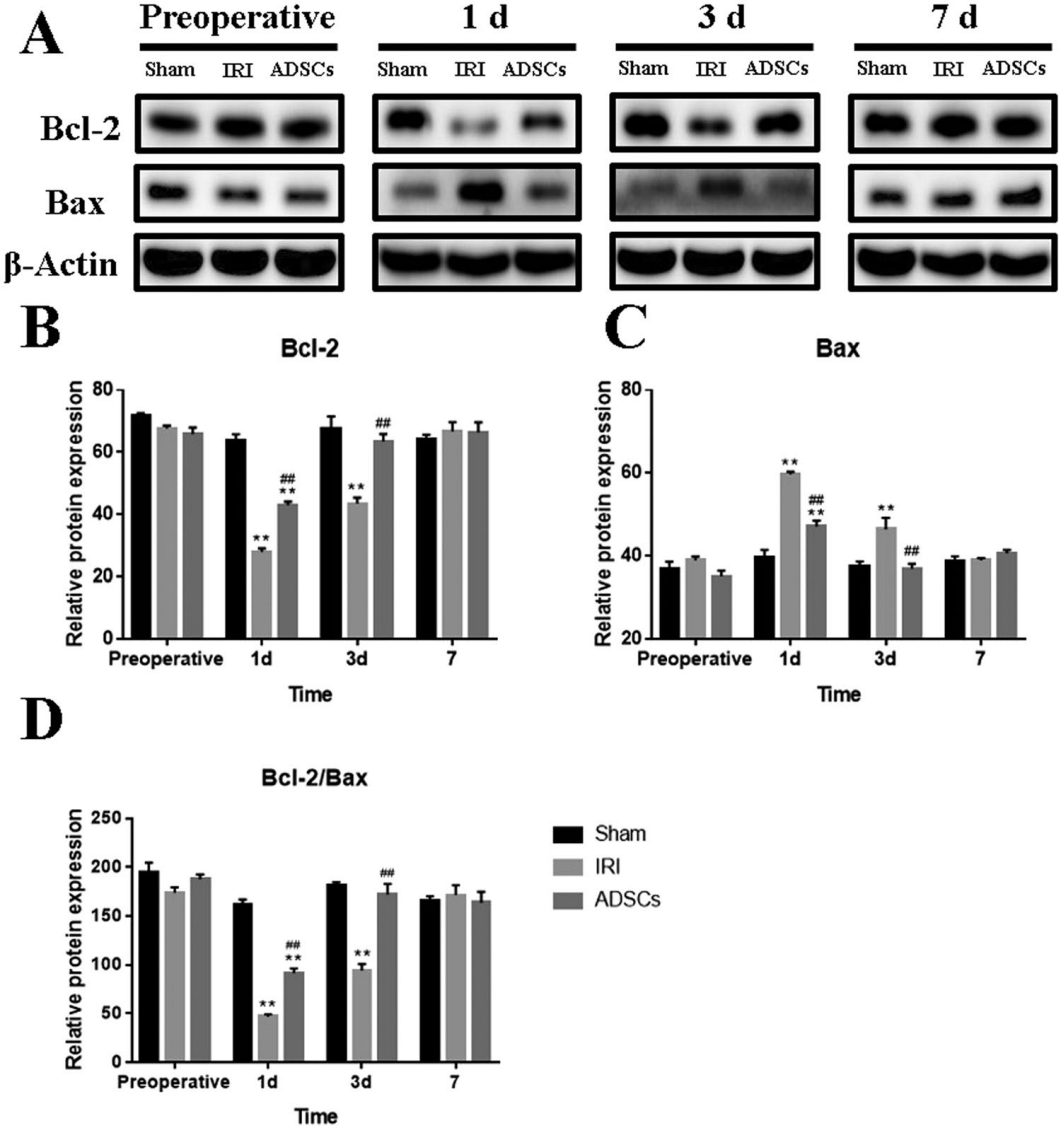
Apoptosis protein expression in liver tissues. (**A**) Representative Western blot analysis of Bcl-2, Bax, and β-Actin. (**B**–**D**) Quantification of Bcl-2, Bax, and Bcl-2/Bax ratio. The blots in A were cropped from different parts of the same gel, or from different gels. The corresponding full-length gels are included in the supplementary information file. **P < 0.01, vs. sham group, ^##^P < 0.01, vs. IRI group.

The expressions of Bcl-2 and Bax proteins were assessed via Western Blotting (Fig. 6A). Compared to the Sham group, the expression of Bcl-2 decreased significantly on 1 d in both the IRI and the ADSC groups, and on 3 d in the IRI group (p < 0.01). ADSC treatment significantly increased the expression of the Bcl-2 protein during 1 d and 3 d (p < 0.01) (Fig. 6B). Additionally, the expression of the Bax protein peaked on 1 d and decreased on 3 d (p < 0.01). After treatment with ADSCs, Bax levels significantly decreased on 1 d and 3 d compared to the IRI group (p < 0.01) (Fig. 6C). Moreover, I/R combined with hepatectomy induced a sharp decrease in the ratio of Bcl-2/Bax protein on 1 d and 3 d in the IRI group (p < 0.01). ADSCs treatment significantly increased the ratio between 1 d and 3 d compared to the IRI group (p < 0.01), although the ratio of the ADSC group was significantly lower than that of the Sham group on 1 d (p < 0.01) (Fig. 6D). This finding suggests that ADSCs suppress hepatic apoptosis by regulating the expression of Bcl-2 and Bax proteins.

## Discussion

ADSCs are adult stem cells that are easily obtained via minimally invasive surgery. Therefore, ADSCs have been suggested as an attractive therapeutic tool in regenerative medicine where they have been used to treat a variety of diseases. In this study, ADSCs, harvested from porcine subcutaneous fat, showed the potential to differentiate into hepatocytes *in vitro*. After liver parenchyma transplantation, ADSCs exerted an anti-apoptotic effect against I/R and hepatectomy injury.

The liver is the largest parenchymal organ in Mammalia, with biological functions such as protein synthesis, detoxification, and glycogen storage. Hepatic I/R injury is one of the key factors during partial hepatectomy, living donor liver transplantation, and trauma; it may cause postoperative morbidity and mortality^22^. Kuo *et al*. confirmed the existence of apoptotic cells in the liver following hepatic I/R, and reported that the number of apoptotic cells correlated positively with the degree of I/R injury^23^. It has been reported that hypoxia, release of ROS, Ca^2+^ overload, and low pH during hepatic I/R could cause a decrease in transmembrane potential, changes in mitochondrial membrane permeability, and destruction of electron transport chains in mitochondria. However, changes in the structure and function of mitochondria will determine the fate of cells^24^. In addition, ROS can directly affect DNA, cause chromosomal aberration of DNA fragmentation, and affect the cell signal transduction^25^. Ca^2+^ overload can degrade DNA by activating Ca^2+^ dependent endonucleases and acting as a second messenger to regulate the expression of transcription factors and apoptosis^26^. Overproduction of ROS caused by I/R injury may induce oxidative damage of macromolecules such as DNA, protein and lipid, and induce apoptosis of hepatocytes^27^. In addition, the massive release of inflammatory mediators caused by I/R injury also aggravates hepatocyte apoptosis^28^. The anti-oxidative and anti-inflammatory effects of ADSCs have been reported^29,30^, which may be a mechanism that indirectly inhibits hepatocyte apoptosis after I/R injury.

When cell apoptosis occurs, inflammatory cells are activated and accumulate in ischemia tissue, thus damaging parenchymal cells. At the same time, both the endoplasmic reticulum and mitochondria expand and chromatin condensation is concentrated, which is accompanied by nuclear membrane shrinkage. In the present study, ADSCs treatment can reduce histopathological and ultrastructural damage, which is supported by previously reported data^31,32^. Furthermore, TUNEL results showed a similar trend: the apoptosis-positive cells were significantly reduced after ADSC treatment.

Fas and its ligand FasL are the main receptor pathways that induce apoptosis. When Fas is combined with FasL, this first induces Fas to form a trimer form, capable of transmitting signals. Subsequently, the death domain binds to the death domain related protein, transmitting apoptotic signals to Caspase8. The activation of Caspase8 will generate a series of conjugates that induce apoptosis. Mesenchymal stem cells (MSCs) play a dual role in the regulation of both Fas and FasL. MSCs may induce apoptosis of T lymphocytes by producing FasL; In contrast, MSCs therapy can reduce the apoptosis of Kupffer cells in a non-heart-beating liver transplantation model by reducing the expression of Fas and FasL^33,34^. Our results show that transplantation of ADSCs can significantly inhibit the expression of Fas and FasL proteins as well as mRNA in the liver.

Caspase belongs to the family of cysteine aspartic proteases. When Caspase is stimulated by a specific signal, an activation enzyme is formed from the inactive zymogen state, initiating a series of cascade reactions that decompose a variety of proteins, thus promoting apoptosis. The apoptosis-initiating factors Caspase8 and Caspase9 activate cascade downstream Caspase and induce apoptosis. Moreover, Caspase3 is the main apoptotic executioner and its activation is a sign of irreversible apoptosis^35,36^. Recent studies have demonstrated that ADSC treatment inhibited the activation of Caspase3 and Caspase9^37–39^. In our study, ADSC treatment significantly decreased the activities of Caspase3, Caspase8, and Caspase9, which may be due to the reduced Fas and FasL expression by ADSC treatment.

In the signal transduction pathway of apoptosis, the proportion of members of the Bcl-2 family is a key factor in the regulation of apoptosis. Bax and Bcl-2 regulate apoptosis via formation of homo- or heterodimers. Apoptosis is induced when Bax forms a homodimer and when Bax forms a heterodimer with Bcl-2 inhibits apoptosis. The ratio of Bcl-2 to Bax protein determines the ratio of heterodimer (Bcl-2/Bax) to homodimer (Bax/Bax), which plays a key role in determining the susceptibility to apoptosis^40,41^. Qin and Liu’s studies have shown that the transplantation of ADSCs reduced apoptosis in hind limb and cerebral I/R injury models^42,43^. Our study shows that ADSCs treatment reduces apoptosis by increasing Bcl-2, decreasing Bax expression, and increasing the Bcl-2/Bax ratio.

In summary, our study demonstrates that ADSCs were successfully transplanted and survived in the receiver liver, where they exert an anti-apoptosis role in the model of liver injury-induced by I/R and hepatectomy. ADSCs may protect the liver by reducing the expression of Fas and FasL, inhibiting Caspase enzyme activities, and increasing the Bcl-2/Bax ratio. Thus, our findings indicate ADSCs as potential therapeutic agents for hepatic I/R injury and subsequent hepatectomy.

## Electronic supplementary material

Supplementary Information
