# Author Correction: Adipose-derived stem cells alleviate liver apoptosis induced by ischemia–reperfusion and laparoscopic hepatectomy in swine

**DOI:** 10.1038/s41598-022-08060-z

**Published:** 2022-03-14

**Authors:** Yansong Ge, Qianzhen Zhang, Hui Li, Ge Bai, Zhihui Jiao, Hongbin Wang

**Affiliations:** College of Veterinary Medicine, Northeast Agricultural University, Harbin, 150030 People’s Republic of China

Correction to: *Scientific Reports* 10.1038/s41598-018-34939-x, published online 15 November 2018

The original version of this Article contained errors.

In Figure 3, the image used for Figure 3D was incorrect. The original Figure 3 and accompanying legend appears below.

**Figure 3:**
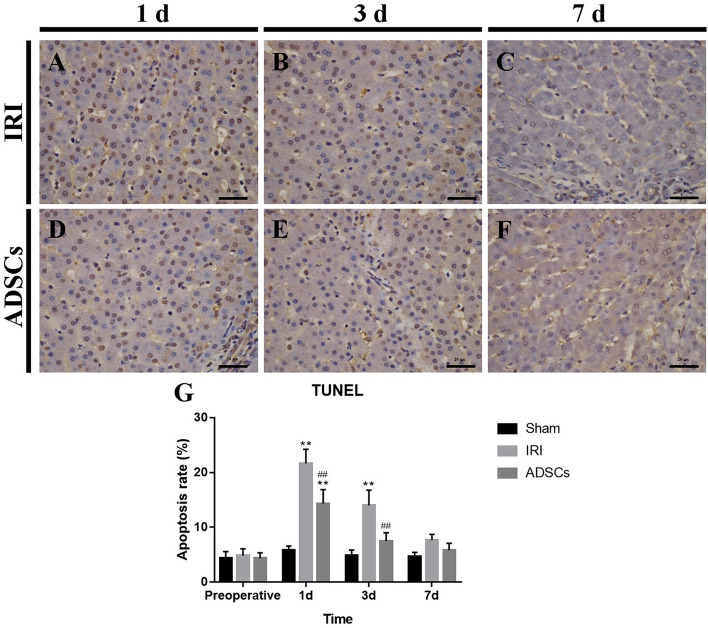
TUNEL staining for liver tissues. (**A**–**C**) IRI group, 1 d, 3 d, and 7 d. (**D**–**F**) ADSCs group, 1 d, 3 d, and 7 d (magnification × 400). (**G**) Apoptosis rate of hepatocytes. **P < 0.01, vs. sham group, ^##^P < 0.01, vs. IRI group.

In Figure 5, the image for Figure 5F was inadvertently duplicated from Figure 3F, and is therefore incorrect. The original Figure 5 and accompanying legend appears below.

**Figure 5:**
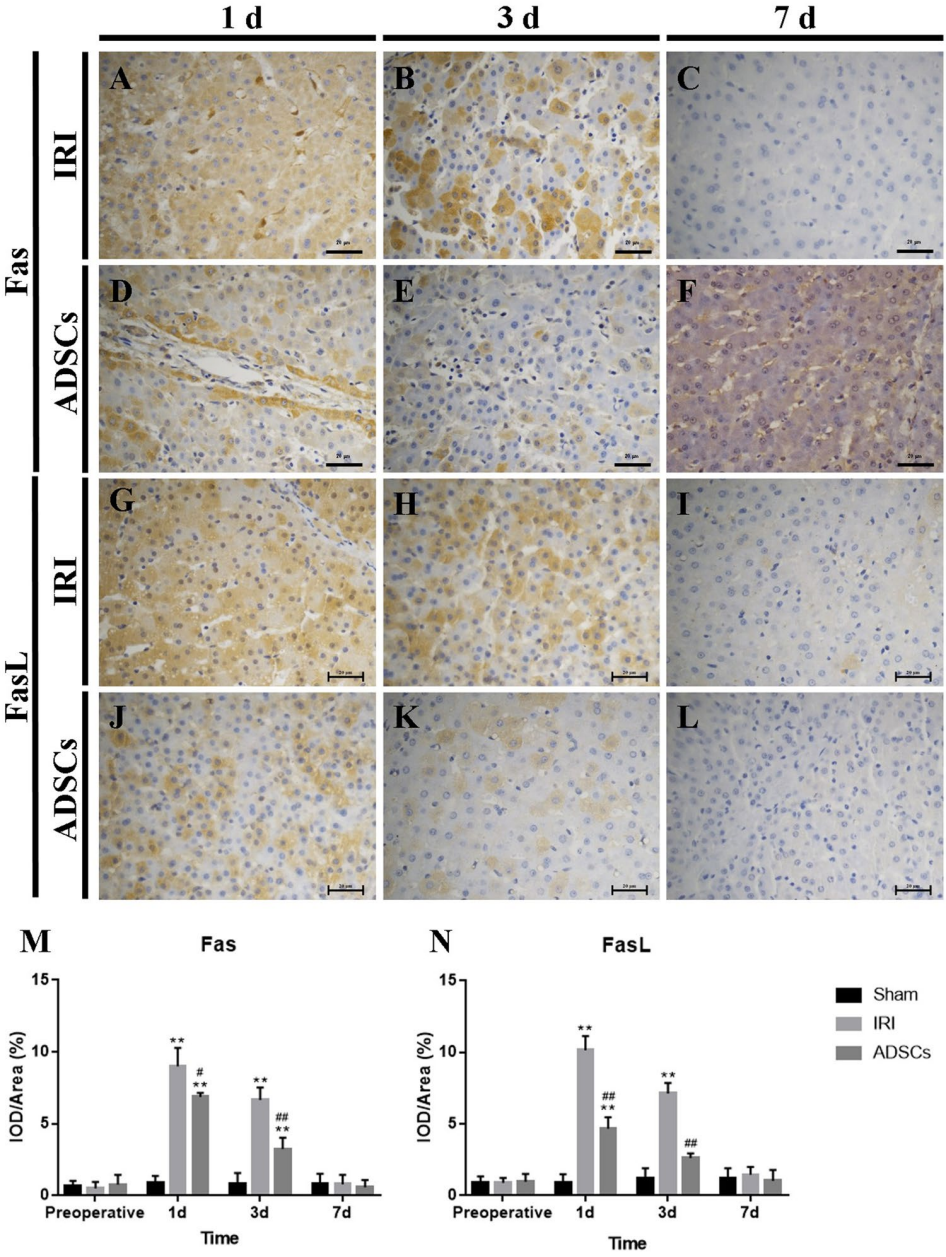
Fas and FasL immunohistochemistry staining of liver tissues. (**A**–**C**) Fas immunohistochemistry staining of the IRI group, 1 d, 3 d, and 7 d. (**D**–**F**) Fas immunohistochemistry staining of the ADSCs group, 1 d, 3 d, and 7 d. (**G**–**I**) FasL immunohistochemistry staining of the IRI group, 1 d, 3 d, and 7 d. (**J**–**L**) FasL immunohistochemistry staining of the ADSCs group, 1 d, 3 d, and 7 d (magnification × 400). (**M** and **N**) Fas and FasL expressions in liver tissues. **P < 0.01, vs. sham group, ^#^P < 0.05, vs. IRI group, ^##^P < 0.01, vs. IRI group.

The original Article has been corrected.

