# Supplementary material for: Adipose-derived stem cells alleviate liver apoptosis induced by ischemia-reperfusion and laparoscopic hepatectomy in swine
Source: Sci Rep. 2018 Nov 15;8:16878. doi: 10.1038/s41598-018-34939-x (PMC6237819; doi:10.1038/s41598-018-34939-x)
Supplement: Supplementary file 1 — Supplementary Information [file 41598_2018_34939_MOESM1_ESM.pdf]

Adipose-derived stem cells alleviate liver apoptosis induced by ischemia-reperfusion and laparoscopic hepatectomy in swine

Yansong Ge[1], Qianzhen Zhang[1], Hui Li[1], Ge Bai[1], Zhihui Jiao[1], Hongbin Wang[1]*

[1]College of Veterinary Medicine, Northeast Agricultural University, Harbin 150030, P.R. China

*Corresponding author.

Address: College of Veterinary Medicine, Northeast Agricultural University, Harbin, 150030, P.R. China

Tel.: +86-451-55190470

E-mail address: hbwang1940@163.com

Full unedited gel for Figure 6 A

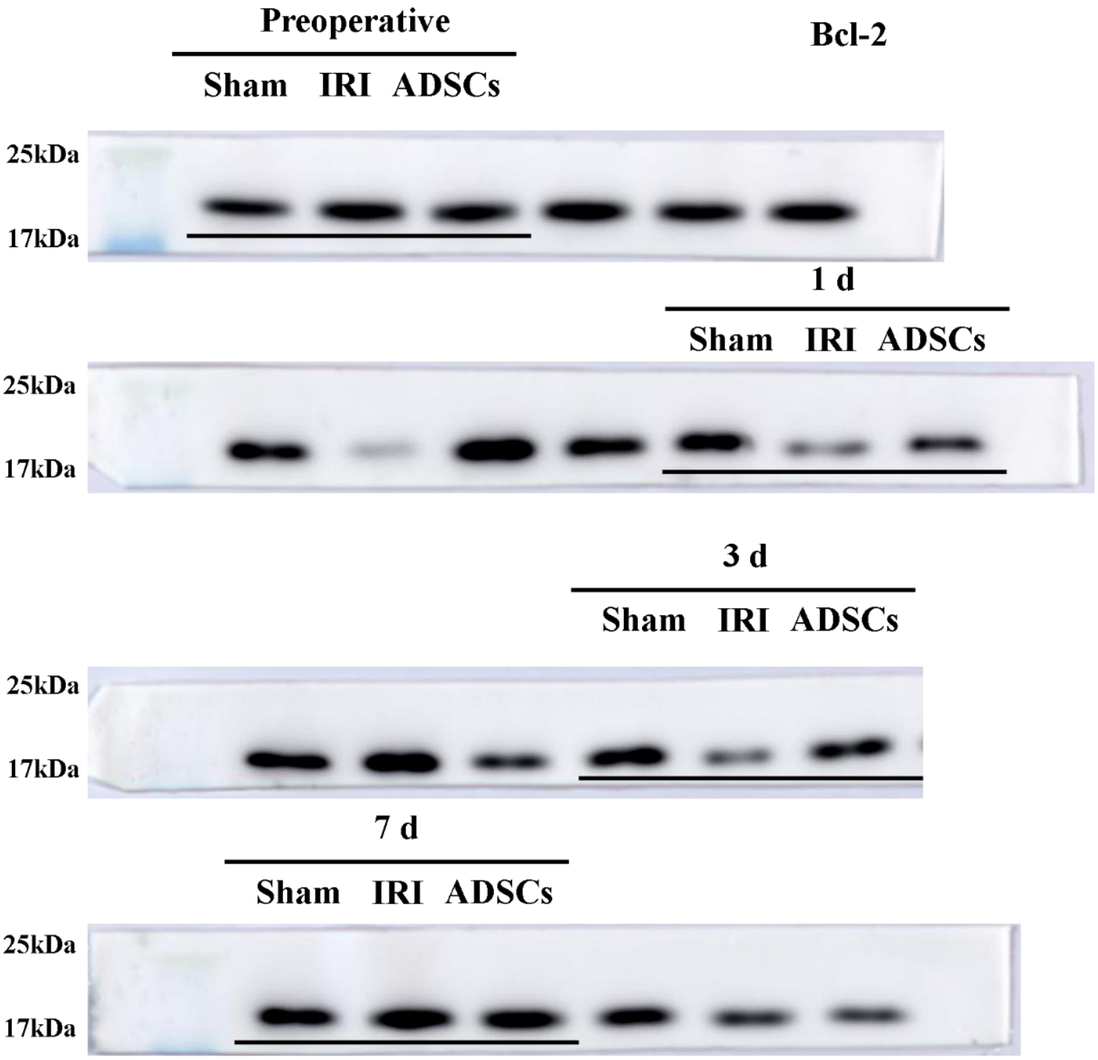

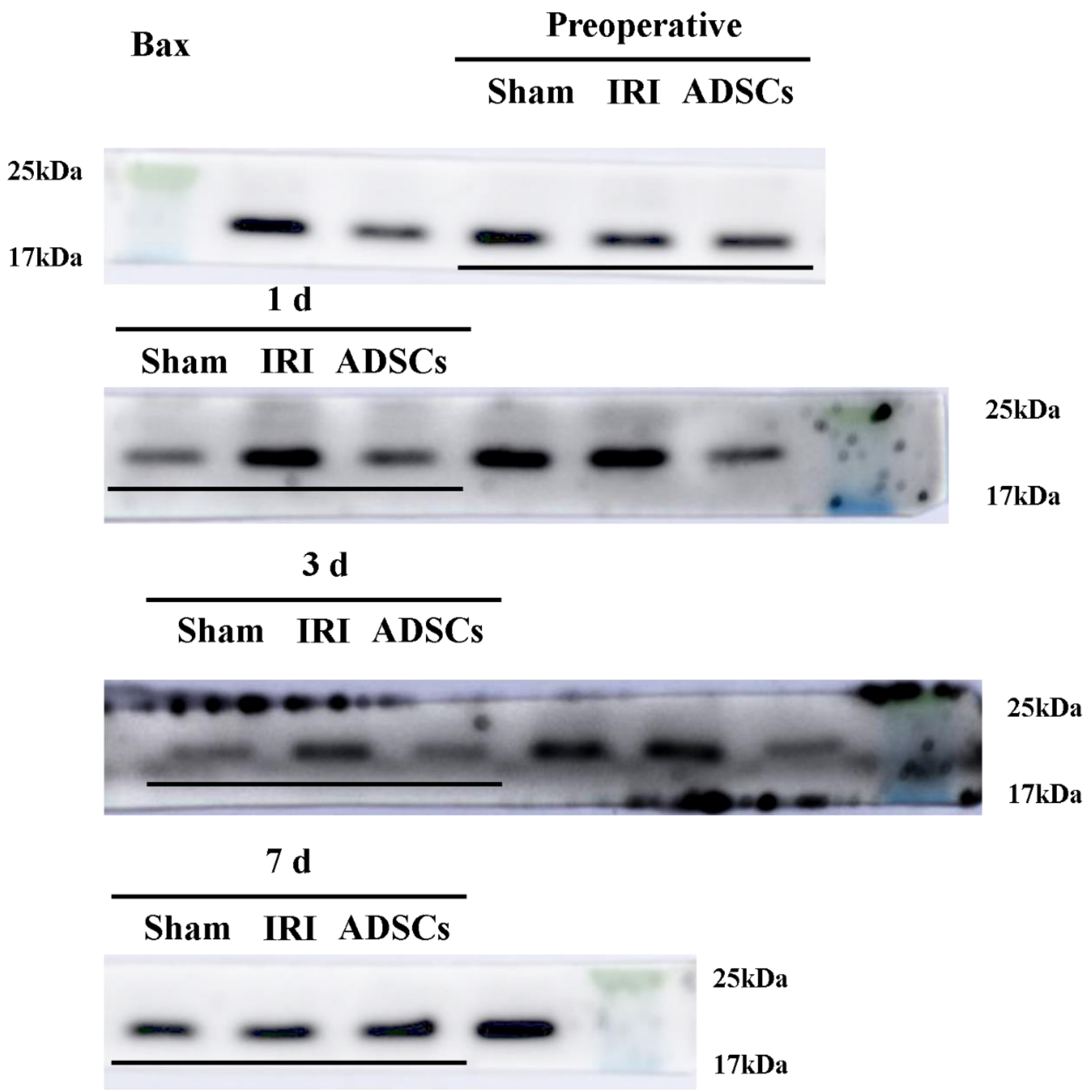
Bax
Preoperative
Sham
IRI
ADSCs
25kDa
17kDa
1 d
Sham
IRI
ADSCs
25kDa
17kDa
3 d
Sham
IRI
ADSCs
25kDa
17kDa
7 d
Sham
IRI
ADSCs
25kDa
17kDa

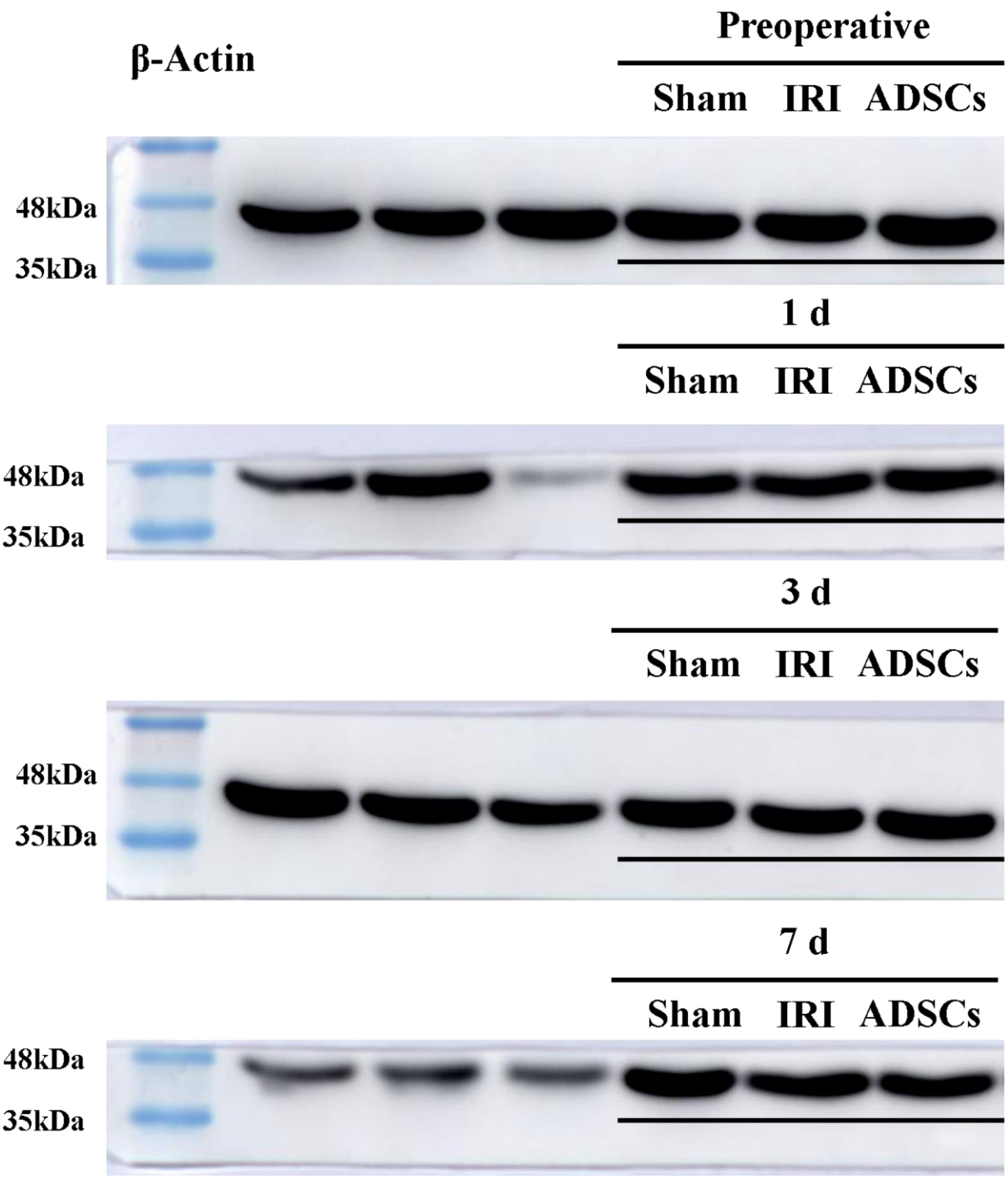
β-Actin
Preoperative
Sham IRI ADSCs
48kDa
35kDa
1 d
Sham IRI ADSCs
48kDa
35kDa
3 d
Sham IRI ADSCs
48kDa
35kDa
7 d
Sham IRI ADSCs
48kDa
35kDa
